# Estimation of root inclination of anterior teeth from virtual study models: accuracy of a commercial software

**DOI:** 10.1186/s40510-019-0298-5

**Published:** 2019-11-22

**Authors:** Panagiota Magkavali-Trikka, Demetrios J. Halazonetis, Athanasios E. Athanasiou

**Affiliations:** 1 Hamdan Bin Mohammed College of Dental Medicine, Mohammed Bin Rashid University of Medicine and Health Sciences, Dubai, United Arab Emirates; 2 Department of Orthodontics, School of Dentistry, National and Kapodistrian University of Athens, Athens, Greece; 3 Department of Dentistry, School of Medicine, European University Cyprus, Nicosia, Cyprus

**Keywords:** Digital models, Natural teeth, Dry skulls, Tooth root inclination prediction software

## Abstract

**Background:**

The aim of the study was to assess the accuracy of commercially available software in estimating anterior tooth root inclination from digital impressions of the crowns of the teeth.

**Subjects and methods:**

Following sample size calculation and application of inclusion and exclusion criteria, 55 anterior natural teeth derived from 14 dry human skulls were selected. Impressions were taken and plaster study models were fabricated. Plaster models were scanned using the high-resolution mode of an Ortho Insight 3D laser scanner. The teeth on the digital scans were segmented and virtual roots were predicted and constructed by the Ortho Insight 3D software. The 55 natural teeth were removed from the dry skulls and scanned using the Identica extraoral white-light scanner in order to calculate their actual root angulation. The teeth were scanned twice, once to acquire the crown and the cervical part of the root, and a second time to acquire the remaining part of the root, including the apex. The two scanned segments were joined in software by superimposing them along their common part. The accuracy of the digital models generated by the Ortho Insight 3D scanner in predicting root angulation was assessed by comparing these results to the corresponding measurements of the 55 natural teeth. The long axes of the tooth models obtained from the software prediction and the scanning of the actual teeth were computed and the discrepancy between them was evaluated. The error of the methods was evaluated by repeating the measurements on 14 teeth and showed an acceptable range.

**Results:**

The predicted tooth angulation was found to differ significantly from the actual angulation, both statistically and clinically. The angle between the predicted and actual long axes ranged from 2.0 to 37.6°(average 9.7°; median 7.4°). No statistically significant difference was found between tooth categories.

**Conclusions:**

Further investigations and improvements of the software are needed before it can be considered clinically effective.

## Background

Compared to traditional plaster study casts, digital models present numerous advantages in terms of cost, time and storage space, without sacrificing measurement accuracy, reliability, and reproducibility [14]. Such models are obtained by intraoral scanning, or from dental impressions and stone casts, but they include only the clinical crowns of the teeth with no root information [12]. Digital models of the whole teeth can be acquired by segmenting CBCT data. However, due to the limited resolution of CBCT images and accompanying image artefacts, such models lack high detail [5, 9, 10, 17]. Combining the two technologies makes it possible to fuse the highly detailed scanned crown surfaces with the CBCT root surfaces into one virtual whole tooth model [17]. Nevertheless, this requires radiation exposure that should be avoided [11]. An alternative is to estimate the shape and position of the roots solely from the crown position and morphology. The Ortho Insight 3D™ laser scanner, in conjunction with the Motion View Software, LLC (Chattanooga, Tennessee, USA) provides estimates of the roots using crown-based algorithmic prediction. The accuracy of the models generated using this commercially available software was tested by comparing the results to actual data obtained from CBCT images [8]. The maximum angular disparity between the long axes of the estimated virtual roots and the actual roots derived from CBCT data reached 40°. The upper and lower canines produced the worst results, followed by the lower lateral incisors. The upper central incisors showed the best results, although the maximum angular difference exceeded 20°, with the median around 8°.

Since CBCT images have limitations in accurately measuring crown and root lengths [2, 7, 16], evaluating software-estimated root angulation would be more valid if data derived from natural dental units were used. Therefore, our hypothesis was that there are differences between the actual root angulation of anterior teeth and the angulation estimated by commercially available software. Our aim was to assess the accuracy of software-generated digital models of anterior teeth by comparing the predicted root angulation to data obtained from natural teeth on dry skulls.

## Methods

### Sample

The sample consisted of 55 natural anterior teeth derived from 14 dry human skulls (11 mandibles and 3 maxillae). The inclusion criteria were that all the teeth should derive from permanent dentition of adults and should present normal crown morphology. Primary teeth, teeth with abnormal tooth morphology and restorations, and jaws resembling craniofacial anomalies or syndromes were excluded. Only anterior teeth were evaluated since they are simpler in terms of the morphology of both their crowns and roots [7]. The number and distribution of the 55 teeth are presented in Table 1.

**Table 1:** Number and distribution of the 55 teeth of the sample

	Maxilla	Mandible
Right central incisor	2	8
Left central incisor	1	6
Right lateral incisor	1	7
Left lateral incisor	2	6
Right canine	2	9
Left canine	2	9
Total	10	45

### Methods

Plaster models were constructed from alginate impressions of the dry skull jaws. Each plaster model was scanned by the first author using the Ortho Insight 3D laser scanner and software (Motionview Software LLC, Chattanooga, Tennessee, USA, software version 4.0.6), with the scanning resolution set at “high”. According to the manufacturer’s instructions, the operator placed landmarks on the digital models (eight points on each incisor and three points on each canine) and identified the facial axes of the teeth. Using this information, the software constructed virtual roots. The tooth models, including the roots, were exported as stereolithography (STL) files.

**Fig. 1:**
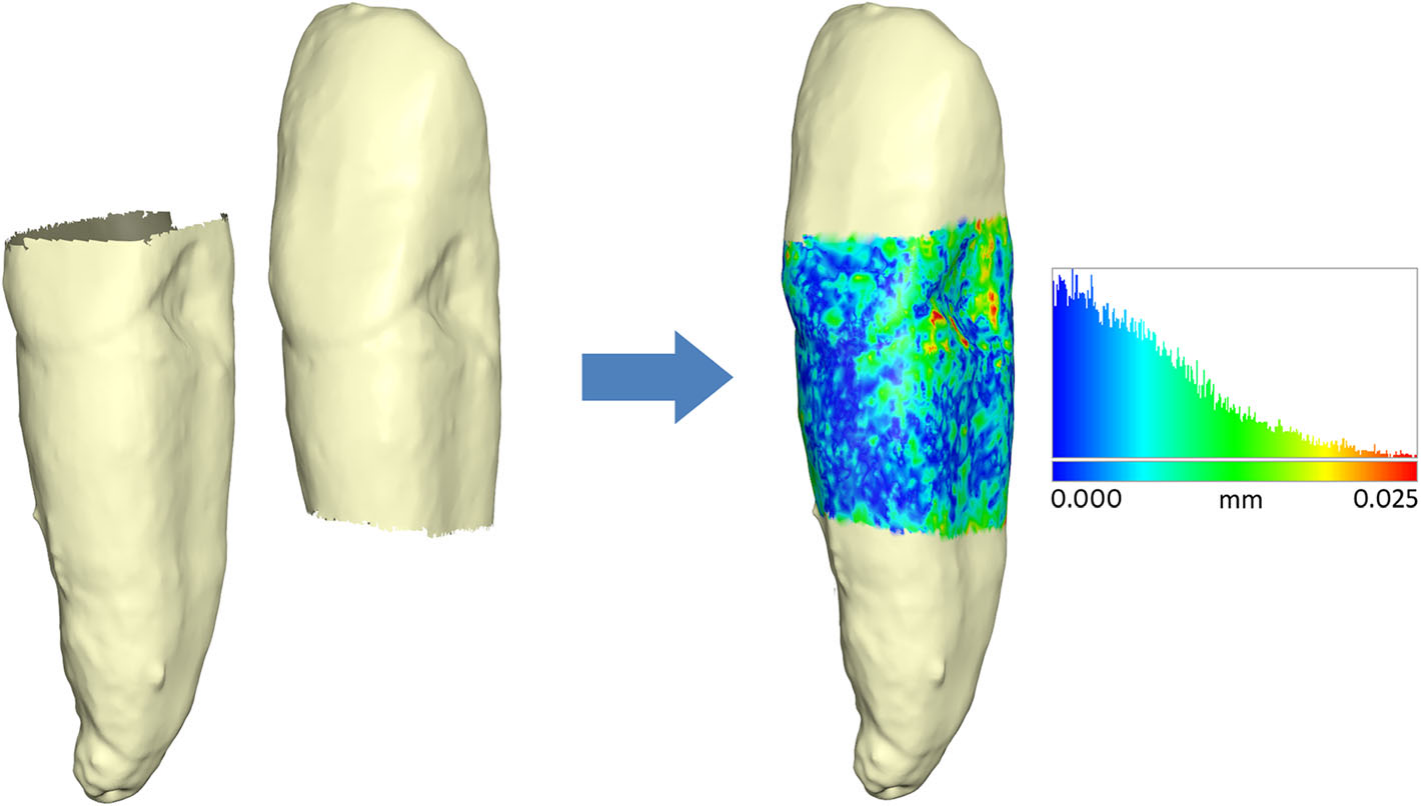
Superimposition of the tooth segments to construct a single tooth model. The colour map of the overlapping areas shows the absolute distances between the meshes at each mesh vertex. The histogram shows that most of the distances were below 0.01 mm, signifying excellent registration

All the 55 natural teeth were removed with care from the dry skulls, covered with a thin layer of white varnish in order to make them suitable for scanning, and scanned using an extraoral white-light scanner (Identica, Medit Co. Ltd, Seoul, South Korea). The teeth were scanned in two stages in order to capture the whole surface: the first stage included scanning the crown and the cervical part of the root, extending as far apically as the retention base of the scanner would allow; at the second stage, the tooth was turned over and the whole root was scanned together with part of the crown. The two segments of each tooth were then superimposed over their common surface to create a single tooth model (Fig. 1).

**Fig. 2:**
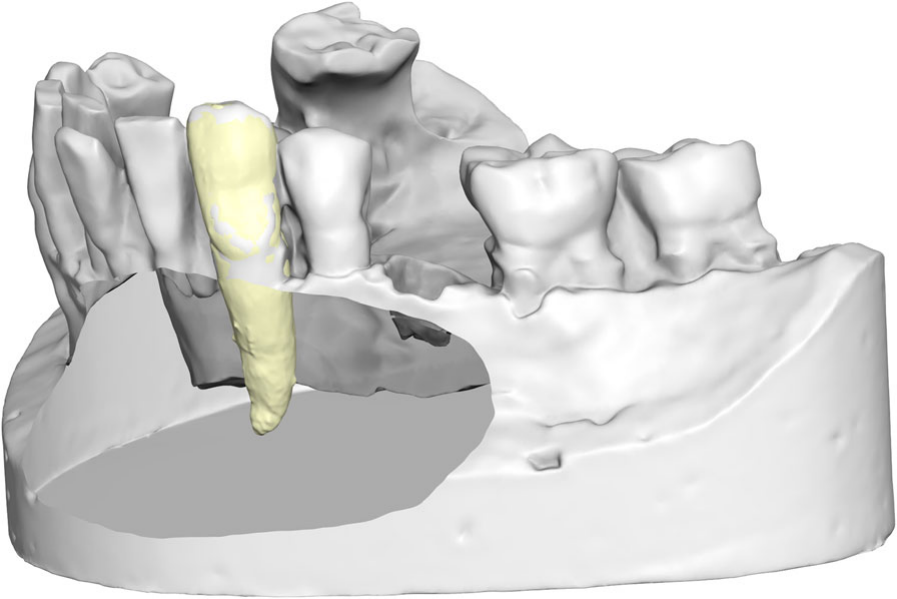
Superimposition of the tooth model on the digital cast, using the crown for alignment. The cast has been cut to reveal the root of the canine

Each unified tooth model was then aligned on the respective tooth of the scanned plaster cast fabricated from the impressions taken from the dry human skulls. The reference area for this superimposition was the part of the crown visible on the casts. In most cases, the cemento-enamel junction was visible on the casts, so the whole crown was used, except for the contact areas to neighbouring teeth (Fig. 2).

**Fig. 3:**
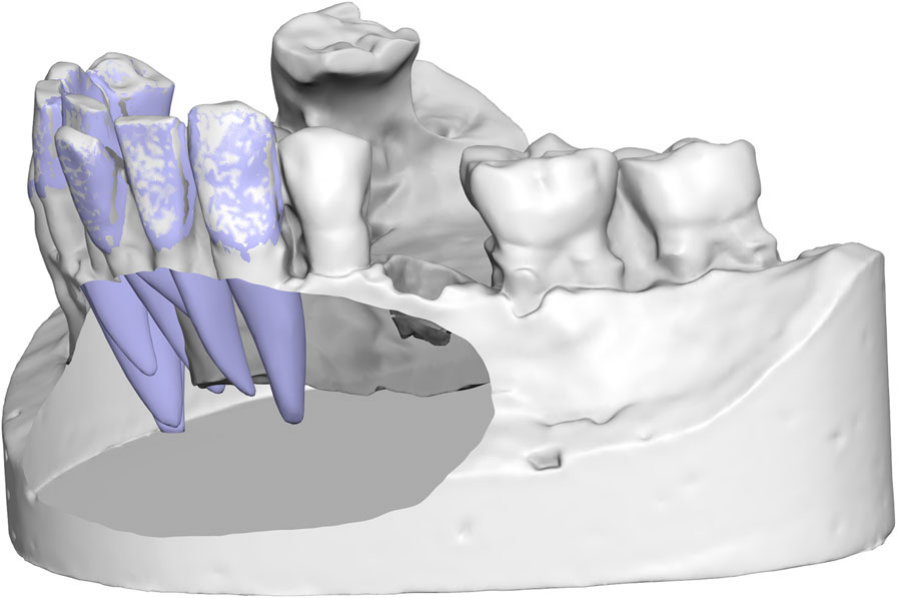
Superimposition of the teeth and roots, as estimated by the software, on the digital cast. Only the crowns were used for the alignment. The cast has been cut to reveal the estimated roots

The third superimposition was between the scanned plaster cast and the Ortho Insight prediction. More specifically, the Ortho Insight prediction included all the teeth of each jaw, joined together in one model. The crowns of these predicted teeth and the corresponding cast crowns were used as reference areas for the superimposition (Fig. 3).

All superimpositions were performed with the Viewbox software (dHAL Software, Kifissia, Greece) using a version of the iterative closest point (ICP) algorithm [3, 6, 15]. The quality of all superimpositions was assessed by the root-mean-squared distance (mm) (RMSD), computed as the square root of the average of the squared distances of the points of one of the surfaces to the closest point on the other surface.

**Fig. 4:**
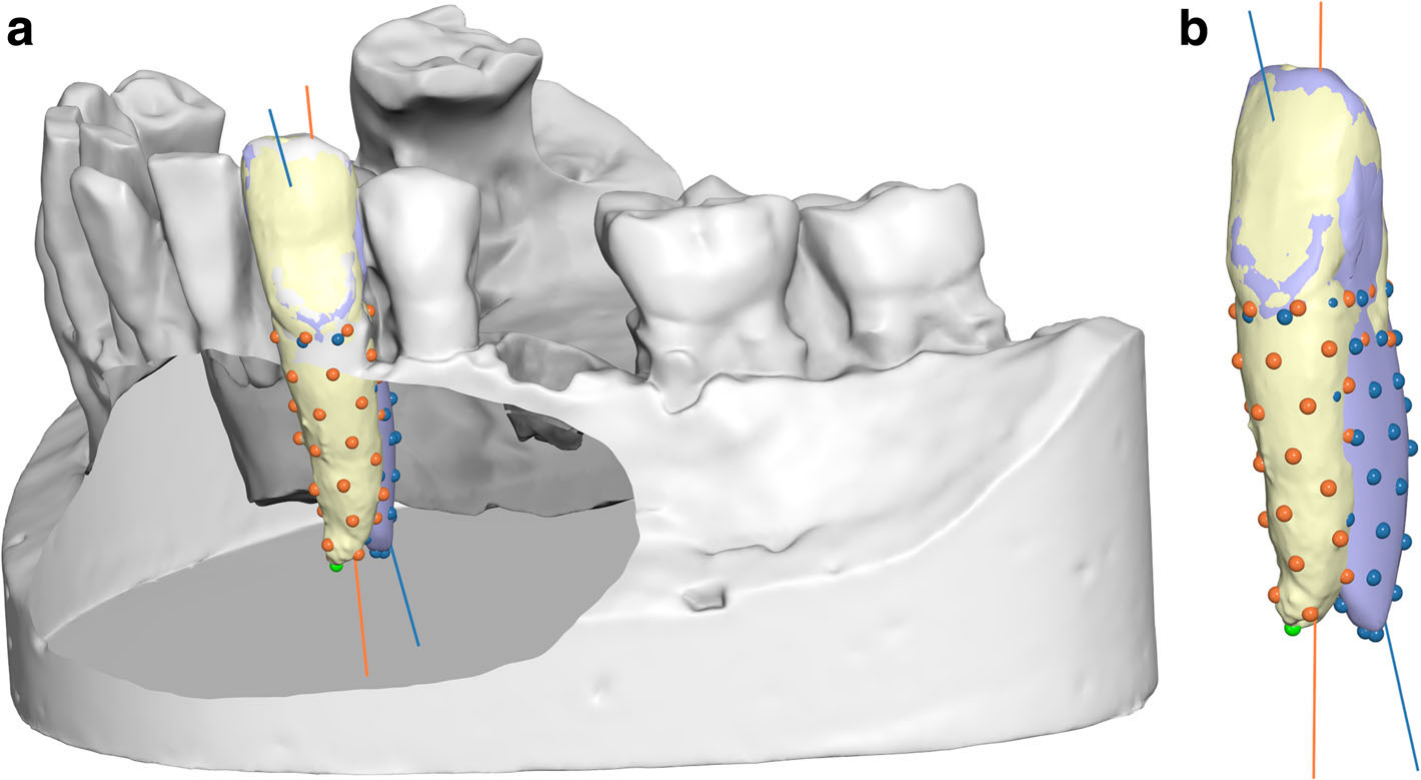
The long axes of the actual and estimated roots of the canine, as computed by the Viewbox software. The dots on each root represent points evenly distributed on each root (50 points per root). The lines are best-fit lines to these points

The second part of the laboratory work consisted of landmarking the virtual roots, both the actual and estimated, to identify their long axes. The Viewbox software automatically placed 50 points uniformly distributed over the root surface of each tooth; the root long axis was computed as the best fit line to these points. Subsequently, the angle between the long axes of the actual tooth root and the estimated tooth root was computed (Fig. 4).

### Sample size calculation

We computed the required sample size based on the requirement to detect a difference of 3° between the actual root angulation and that estimated by the software. We set the alpha level to 0.05, power to 80% and the standard deviation of the difference to 7°, based on a previous study [8]. These values resulted in a minimum sample size of 43 teeth; we included 55, based on availability (Table 1).

### Statistical methods

Data were entered in an Excel file (Microsoft Corp., Redmond, Washington, USA) and SPSS for Windows (version 20.0, SPSS Inc., Chicago, Illinois, USA) was used for the representation of the data and numerical representation of the difference in root angulation between the actual tooth and its prediction.

### Method error

In order to evaluate intra-examiner error, 14 teeth were randomly selected and their scanning was repeated by the first author after an interval of at least 1 month. The alignment of the crown and the root to construct a whole-tooth model was repeated and the RMSD was re-computed. The angle between the long axis of the rescanned tooth and the prediction was re-estimated. The 95% limits of agreement (LoA) were computed to assess intra-examiner error [4].

## Results

### Method error

The difference in RMSD between the two repeated trials for the overlap between the crown and the root ranged from –0.006 to 0.010 mm (mean 0.001 mm, 95% LoA –0.007 to 0.010 mm), indicating acceptable reliability. Regarding the overlap between the tooth and the cast, RMSD differences ranged from –0.040 to 0.057 mm (mean –0.002 mm, 95% LoA –0.049 to 0.045 mm), also showing good repeatability. No systematic error was detected in the repeated angular measurements between the estimated and the actual root angulation (mean value of the difference between repeated measurements: –0.2°, 95% LoA –3.69 to 3.18°).

### Quality of superimpositions

**Fig. 5:**
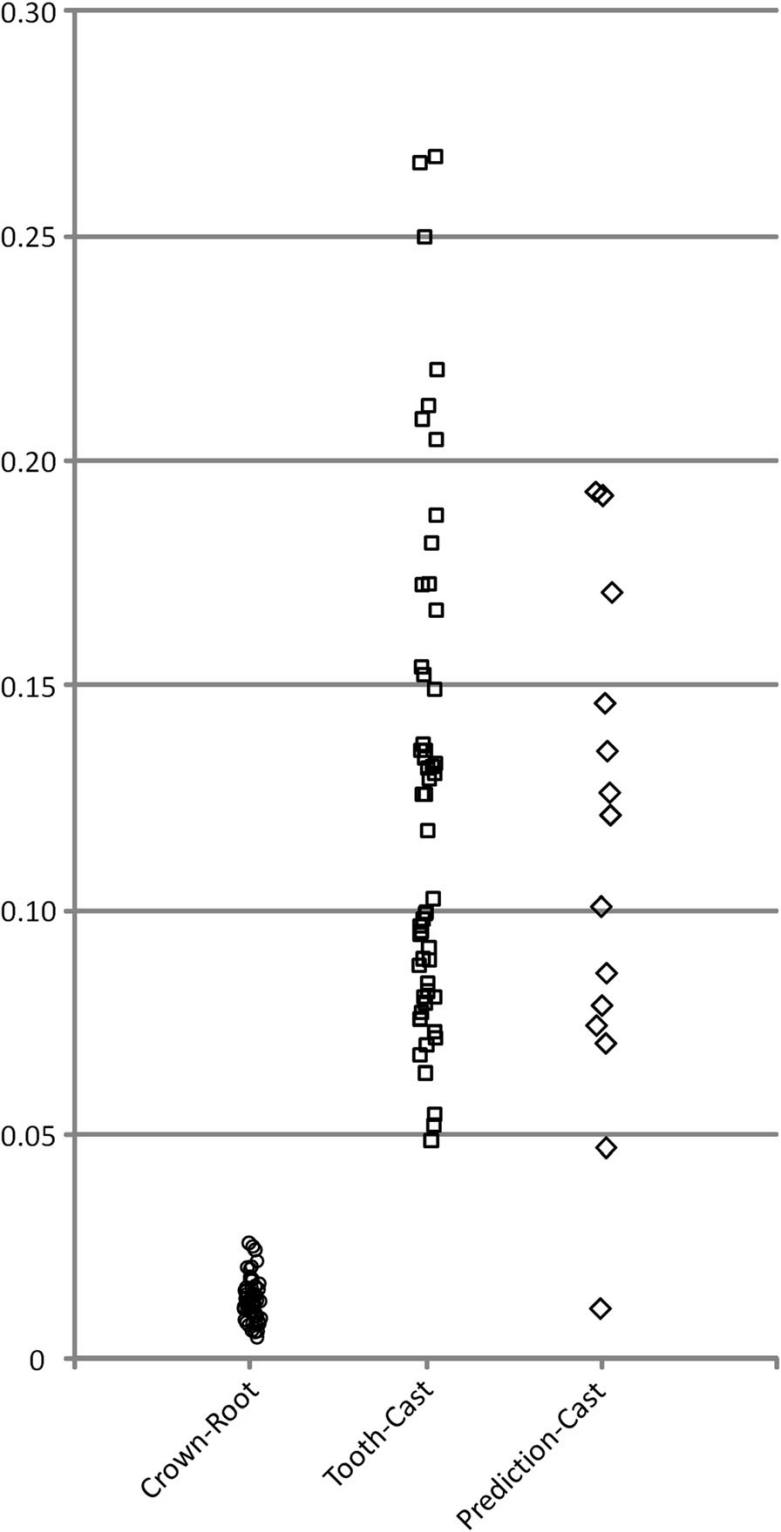
RMSD values (mm) for each superimposition type

The crown-root superimposition of the tooth segments was reliable and accurate, since the minimum value of the RMSD was 0.005 mm, with the highest being 0.026 mm (median 0.013). The superimposition between each scanned copy of the 3D tooth with the corresponding tooth of the scanned cast showed an RMSD ranging from 0.048 to 0.267 mm (median 0.103). The RMSD of the superimposition between the dry skull casts and the prediction estimated by the software ranged from 0.011 to 0.193 mm (median 0.111) (Fig. 5).

### Difference in angulation between actual and estimated roots

**Fig. 6:**
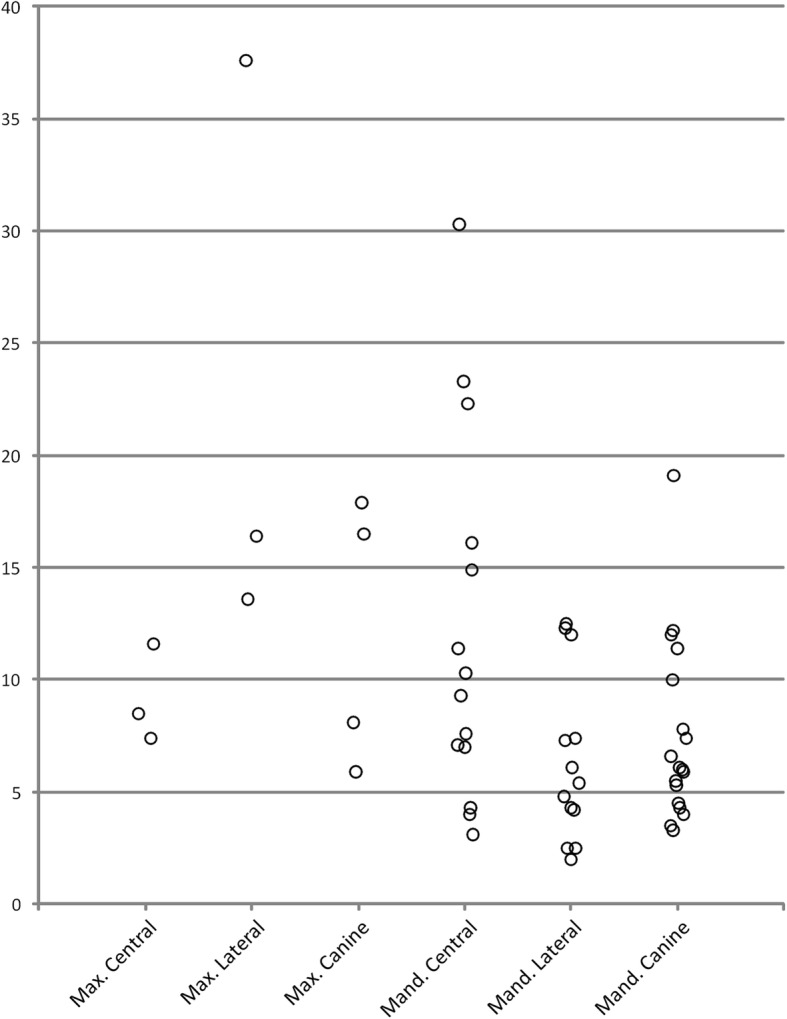
Differences in angulation (degrees) for each tooth category

Descriptive statistics for the angle between the long axis of the actual and the predicted root for each tooth category are shown in Table 2. The minimum value was 2.0°, corresponding to a lower left lateral incisor, and the maximum angle was 37.6°, corresponding to an upper left lateral incisor. Fig. 6 shows a plot of the differences in angulation for each tooth category (Additional file 1).

**Table 2:** Descriptive statistics of the angle (degrees) between the long axis of the actual and the estimated root

	*n*	Mean (SD)	Median (range)
Maxilla			
Central incisor	3	9.2 (2.18)	8.5 (7.4 to 11.6)
Lateral incisor	3	22.5 (13.12)	16.4 (13.6 to 37.6)
Canine	4	12.1 (5.98)	12.3 (5.9 to 17.9)
All maxillary teeth	10	14.4 (9.21)	12.6 (5.9 to 37.6)
Mandible			
Central incisor	14	12.2 (8.20)	9.8 (3.1 to 30.3)
Lateral Incisor	13	6.4 (3.74)	5.4 (2.0 to 12.5)
Canine	18	7.5 (4.06)	6.1 (3.3 to 19.1)
All mandibular teeth	45	8.6 (6.01)	7.0 (2.0 to 30.3)
Overall	55	9.7 (6.96)	7.4 (2.0 to 37.6)

A Kruskal-Wallis test on the angles of the mandibular teeth did not reveal any evidence of a statistically significant difference between the three categories—central incisor, lateral incisor and canine (*P* = 0.097).

The visual observation of the cases revealed that the software frequently estimated angulations that resulted in overlapping of adjacent roots, a clinically impossible situation unless there is extensive root resorption or root morphology variation.

## Discussion

The increasing penetration of digital technology in orthodontic practice has opened up new prospects for hardware and software development. The reliable estimation of root position from dental arch scans is a worthy goal, as it avoids radiation exposure. At present, we are aware of only one software company that markets such a tool. This has been evaluated in a previous study, which showed a large range of discrepancy in the angle between the actual and estimated roots, reaching almost 40° in extreme cases [8]. However, CBCT images were used to acquire root information; since crown and root lengths derived from CBCT images may not be accurate due to resolution issues and artefacts [2, 7, 16], testing the software would be more appropriate with data derived from natural teeth.

In this investigation, we tested for software-estimated root angulation using actual teeth from dry human skulls. The research hypothesis was verified because significantly different angulations between the estimated and the actual roots were clearly demonstrated. The median discrepancy of the estimated angulation was 7.4 , with three teeth, namely two lower central incisors and one upper lateral incisor, displaying extreme values of 23.3, 30.3 and 37.3 of difference in angulation, respectively. The minimum values were found in two cases of lower lateral incisors (2.0 and 2.5 ). The errors of 10 or more in estimating mesio-distal or labio-lingual angulation of the roots are considered clinically significant, since in these cases the parallelism is characterized as poor [13]. Visual observation of the virtual models revealed several cases where the estimated roots overlapped with one another or extended outside the physical limits of the dental cast. The great variation in predicting root angulation observed in this study raises serious concerns about applying the software in the clinical environment.

The present study was limited to anterior teeth since preliminary visual evaluation of the digital models indicated that the crowns and roots of these dental units are much better defined than the posterior. We restricted our evaluation to root angulation and did not measure other important morphological features, such as root length, volume and shape. Furthermore, the method did not assess the direction of the angulation discrepancy, i.e., whether it was mesio-distal or labio-lingual.

The limitations of the study include the use of alginate vs. a higher accuracy silicone material for the impressions, the use of varnish, albeit of small thickness, to coat the teeth for reliable scanning, and the need for three separate superimpositions, each with its own inherent errors. However, the overall error induced by these factors is expected to be small compared to the observed discrepancies in estimated root angulation.

The software tested here has been positively evaluated regarding routine clinical orthodontic applications, such as space analysis and tooth size discrepancy assessment [1]. Estimation of root morphology and position is not one of its primary functions and our results confirm that further development is needed to reach acceptable validity. However, the aim of estimating root position solely from crown information is a worthy goal, as it eliminates the need for radiation exposure. Accurately estimating root position may improve quality of treatment; biomechanical adjustments of fixed appliances, appropriate attachment design in clear aligner therapy and safe placement of temporary anchorage devices are a few examples of the potential positive outcomes.

## Conclusions

The results of the study lead to the following conclusions:

The angle between true root position and estimated position ranged from 2 to 37.6° and the mean value was 9.7°.

Visual observation of the cases showed that the software frequently estimates angulations that create an overlapping of adjacent roots, a clinically impossible situation unless there is extensive root resorption or root morphology variation.

Further investigations and improvements of the software are needed before it can be considered useful for routine clinical use.

## Supplementary information

**Additional file 1.** Angle between the long axis of the actual and the estimated root of each tooth. Tooth type: 1: central incisor, 2: lateral incisor, 3: canine. Jaw: 1: maxilla, 2: mandible.

## Data Availability

The datasets used and/or analysed during the current study are available from the corresponding author on reasonable request.

## Abbreviations

CBCT: Cone beam computed tomography
ICP: Iterative closest point
LoA: Limits of agreement
RMSD: Root-mean-squared distance
SPSS: Statistical Package for the Social Sciences
STL: Stereolithography
