# Supplementary material for: Estimation of root inclination of anterior teeth from virtual study models: accuracy of a commercial software
Source: Prog Orthod. 2019 Nov 22;20:43. doi: 10.1186/s40510-019-0298-5 (PMC6872682; doi:10.1186/s40510-019-0298-5)
Supplement: Supplementary file 1 — Additional file 1. Angle between the long axis of the actual and the estimated root of each tooth. Tooth type: 1: central incisor, 2: lateral incisor, 3: canine. Jaw: 1: maxilla, 2: mandible. [file 40510_2019_298_MOESM1_ESM.docx]

Additional file 1

Angle between the long axis of the actual and the estimated root of each tooth. Tooth type: 1: central incisor, 2: lateral incisor, 3: canine. Jaw: 1: maxilla, 2: mandible.

| Tooth | Tooth type | Jaw | Angle (degrees) |
| --- | --- | --- | --- |
| Tooth 13122 | 2 | 1 | 37.6 |
| Tooth 05231 | 1 | 2 | 30.3 |
| Tooth 13113 | 3 | 1 | 17.9 |
| Tooth 12243 | 3 | 2 | 11.4 |
| Tooth 05241 | 1 | 2 | 23.3 |
| Tooth 03241 | 1 | 2 | 22.3 |
| Tooth 01233 | 3 | 2 | 6.6 |
| Tooth 06233 | 3 | 2 | 19.1 |
| Tooth 07112 | 2 | 1 | 16.4 |
| Tooth 09241 | 1 | 2 | 16.1 |
| Tooth 07122 | 2 | 1 | 13.6 |
| Tooth 08241 | 1 | 2 | 7.1 |
| Tooth 03233 | 3 | 2 | 10.0 |
| Tooth 03232 | 2 | 2 | 12.3 |
| Tooth 13123 | 3 | 1 | 16.5 |
| Tooth 04241 | 1 | 2 | 10.3 |
| Tooth 05243 | 3 | 2 | 7.4 |
| Tooth 04231 | 1 | 2 | 11.4 |
| Tooth 04232 | 2 | 2 | 12.0 |
| Tooth 03231 | 1 | 2 | 14.9 |
| Tooth 03242 | 2 | 2 | 4.2 |
| Tooth 14111 | 1 | 1 | 11.6 |
| Tooth 05232 | 2 | 2 | 12.5 |
| Tooth 10243 | 3 | 2 | 12.2 |
| Tooth 05242 | 2 | 2 | 7.4 |
| Tooth 07111 | 1 | 1 | 7.4 |
| Tooth 09233 | 3 | 2 | 7.8 |
| Tooth 09242 | 2 | 2 | 7.3 |
| Tooth 05233 | 3 | 2 | 12.0 |
| Tooth 06231 | 1 | 2 | 7.6 |
| Tooth 07113 | 3 | 1 | 8.1 |
| Tooth 02241 | 1 | 2 | 7.0 |
| Tooth 04242 | 2 | 2 | 5.4 |
| Tooth 06241 | 1 | 2 | 4.3 |
| Tooth 04243 | 3 | 2 | 5.9 |
| Tooth 10241 | 1 | 2 | 3.1 |
| Tooth 09243 | 3 | 2 | 6.1 |
| Tooth 10242 | 2 | 2 | 2.5 |
| Tooth 08243 | 3 | 2 | 4.0 |
| Tooth 08231 | 1 | 2 | 4.0 |
| Tooth 08242 | 2 | 2 | 4.3 |
| Tooth 09232 | 2 | 2 | 6.1 |
| Tooth 06243 | 3 | 2 | 3.3 |
| Tooth 07123 | 3 | 1 | 5.9 |
| Tooth 10231 | 1 | 2 | 9.3 |
| Tooth 07121 | 1 | 1 | 8.5 |
| Tooth 08233 | 3 | 2 | 4.3 |
| Tooth 02232 | 2 | 2 | 4.8 |
| Tooth 08232 | 2 | 2 | 2.0 |
| Tooth 02242 | 2 | 2 | 2.5 |
| Tooth 02243 | 3 | 2 | 4.5 |
| Tooth 03243 | 3 | 2 | 6.0 |
| Tooth 02233 | 3 | 2 | 3.5 |
| Tooth 04233 | 3 | 2 | 5.3 |
| Tooth 11233 | 3 | 2 | 5.5 |
